# Rapid phenotypic change in a polymorphic salamander over 43 years

**DOI:** 10.1038/s41598-021-02124-2

**Published:** 2021-11-22

**Authors:** Maggie M. Hantak, Nicholas A. Federico, David C. Blackburn, Robert P. Guralnick

**Affiliations:** Florida Museum of Natural History, University of Florida, Gainesville, FL USA

**Keywords:** Ecology, Evolution

## Abstract

Color polymorphic animals offer a unique system for studying intraspecific phenotypic responses to climate change. Discrete color morphs are easy to identify, and correlated trait responses of morphs can indicate how climate warming may facilitate long-term maintenance of polymorphisms. We use a historical dataset spanning 43 years to examine temporal shifts in color morph frequency and body size in response to climate in the Eastern Red-backed Salamander, *Plethodon cinereus*, which contains a widespread striped/unstriped color polymorphism. We created a pipeline to extract high-throughput trait data from fluid-preserved museum specimens where we batch-photographed salamanders, de-aggregated individual specimens from photographs, and solicited help of community scientists to score color morphs. We used a linear modeling framework that includes information about spatial population structure to demonstrate that color morph frequency and body size vary in response to climate, elevation, and over time, with an overall trend of higher frequency and decreased body size of the striped morph, but increased size of the unstriped morph. These surprising results suggest that morphs may be responding to multiple climate and geographic drivers through co-adapted morphological changes. This work highlights new practices of extracting trait data from museum specimens to demonstrate species phenotypes response to climate change.

## Introduction

While smaller body size commonly correlates with warmer temperatures (i.e., Bergmann’s Rule^1^), the underlying mechanisms remain hotly debated^2^. This trend, when examined spatially, is common in endothermic animals^3,4^ (but see^5^), but is less consistent in ectotherms^6–9^. Still, a set of empirical and theoretical work predicts reductions in body size over time for ectotherms, including the temperature-size rule in which warmer temperatures increase physiological constraints on growth rate, leading to smaller body size^10^. More recent work links this rule with tolerance to heat stress that varies predictably with body size^11^. As such, reductions in body size over time may be a universal response of organisms to climate warming^2^.

Less well-studied are other climate-driven changes, including responses in physiological, behavioral, and other morphological traits^12,13^. Animals with color polymorphisms offer a unique system for studying species-specific responses to climate change. Color pattern is an easily identifiable trait that can be traced across time and space, providing tractable opportunities to study direct effects of climate. Further, color morphs are comprised of co-adapted sets of other phenotypic traits^14^ that suggest morphs occupy divergent fitness optima^15^. Because color morphs have distinct trait optima linked to physiology and thermal ecology, it is possible that climate warming may aid in long-term maintenance of multiple color morphs as niche separation between them increases and competition decreases^16^. While geographic variation in color morph frequency and climate has been well studied^17^, no studies have examined other morphological changes between color morphs in a spatiotemporal context. Inclusion of other ecologically-relevant traits of polymorphic animals, such as body size, can provide information on how morphs adapt to changing environmental conditions.

**Figure 1:**
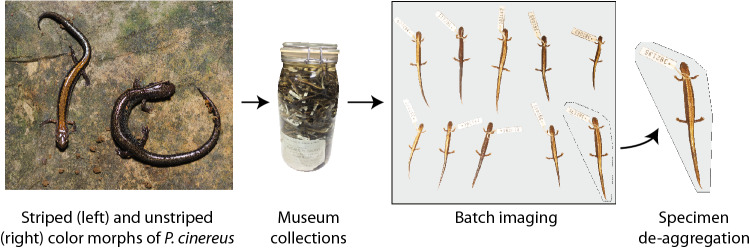
Methodological workflow: striped and unstriped morphs of *Plethodon cinereus* were collected by Richard Highton and deposited into the National Museum of Natural History (NMNH); batch imaging of salamanders (dorsal view) was carried out at the NMNH; subsequently, we de-aggregated specimens from the original image. Individual specimen images were then uploaded to the web-based community science platform Notes from Nature^52^, where volunteers scored color morphs.

Salamanders within the genus *Plethodon* serve as a useful model for examining the effects of climate change, because these lungless, low vagility species require cool, moist microhabitats that facilitate cutaneous respiration^18–21^. Within the North American genus *Plethodon*, the Eastern Red-backed Salamander, *P. cinereus*, presents a particularly good study system for understanding polymorphism in a spatiotemporal framework, as population sizes are large^22,23^, the species is relatively short-lived (estimated lifespan of 5–9 years in wild populations^24^), and is color polymorphic throughout much of its range^25^. There are two common color morphs of *P. cinereus*, a striped morph that has a red stripe overlaid on a black dorsum, and an unstriped morph that is completely black^26^ (Fig. 1). The color morphs are differentiated along several niche axes, including physiology, diet, territoriality, predation responses, and mating interactions^24,27–32^. In addition, the morphs appear to be linked with particular climatic conditions: the striped morph is more often associated with cooler, wetter climatic niches, and the unstriped morph more with warmer, drier conditions^33–35^ (but see^36,37^). Finally, there are conflicting results from studies evaluating the correlation of color morph frequency with shifts in climate^25,38–41^. These studies are difficult to synthesize, as they use different modeling approaches and data sources, and the sampling is conducted over two^41^ or an unknown number of time points^25,39,40^ (the datasets are not publicly available). Whether variation in color morph frequency in *P. cinereus* is associated with other phenotypic changes remains unknown, although changes in morphology (e.g. body size) have important implications for niche use and maintenance of the polymorphism^16^.

Our work focuses on two aspects of phenotypic change between color morphs of *P. cinereus* and their relation to climate. First, we tested whether color morph frequencies shifted over time due to changing climatic conditions. Drawing from previous studies, we predicted an increase in the proportion of unstriped morphs at lower elevation, in warmer areas, and over time as climate has generally warmed across the United States over the last century^42^. Second, we examined whether body size of striped and unstriped morphs diverged over time in response to changes in climate. We predicted striped morphs would decrease in size with warmer temperatures and through time, while unstriped morphs would show no change in size. This prediction is based on three lines of support. (1) The striped morph is less heat tolerant than the unstriped morph^33,34^. (2) The unstriped morph appears to possess more costal grooves – which is correlated with vertebral number^43,44^ and suggests increased fossoriality in salamanders^45–47^. Increased time underground can prevent exposure to unfavorable conditions above ground, and thus, we expect no change in size for unstriped morphs^48^. (3) Caruso et al.^49^ demonstrated body size reductions in *P. cinereus* over a 36-year time period in response to climate change but did not account for color morph. Because the striped morph is more common across the range^40^, the signal of size change may have been driven by the preponderance of striped forms. To address our predictions, we use a unique historical collection by Richard Highton that was deposited at the Smithsonian National Museum of Natural History. Highton’s collections are particularly useful because he amassed time-series of *P. cinereus* across multiple sites, providing spatial control within sites along with replication across sites. In total, we compiled a dataset consisting of 2862 records of *P. cinereus* spanning 43 years (1956–1999) to examine temporal shifts in phenotypic traits of the two distinct color morphs.

## Methods

### Spatial sampling and trait data collection

**Figure 2:**
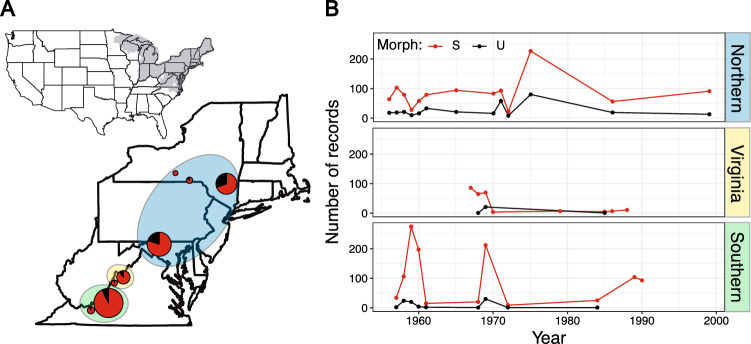
(**A**) The inset map shows (in gray) the range of *Plethodon cinereus* in the United States^96^. Specimen geolocations, separated by less than 15 km, were composited into eight sites. These sites are noted with color morph frequencies (black = unstriped, red = striped); the size of each pie reflects the number of composited *P. cinereus* records from each locality. The colored ellipses around pies indicate the mitochondrial clades to which the sampled localities belong, based on the analysis and mitochondrial clade names from Radomski et al.^50^: blue = Northern clade, yellow = Virginia clade, green = Southern clade. This map was created with R version 3.6.1 (https://www.r-project.org/). (**B**) The number of *P. cinereus* records by morph, collection year (indicated with a point), and mitochondrial clade examined in this study. Color morph and clade colors match A.

Color morph ratios and body size data of *P. cinereus* were obtained from specimens at the Smithsonian National Museum of Natural History (NMNH). We examined 2,988 georeferenced fluid-preserved specimens with a focus on geographic areas with substantial sampling over time (minimum 20-year time series; Fig. 2). In total, specimens were from 37 geolocations. Figure 2 shows relative morph frequencies across eight sites formed by compositing geolocations that were separated by less than 15 km. These sites range from northern Virginia to southern Pennsylvania, and represent three separate mitochondrial clades based on Radomski et al.^50^, as discussed below.

To streamline data collection at the NMNH, we took batch photographs consisting of 8–12 individuals per image of the dorsal and ventral surface of each salamander (Fig. 1). Lighting conditions and background coloration were standardized. Ventral images included a scale bar and a 20 × 25 cm square of plexiglass, 2 mm in depth, which was placed on top of the salamanders to ensure they were flat. We used ImageJ^51^ to collect a measure of body size—snout–vent length (SVL; from the tip of the snout to the anterior end of the cloaca)—of each salamander, unless the cloaca was obscured (e.g. from glare due to plexiglass). In total, we were unable to measure SVL from 85 specimens.

To extract color morph data from dorsal images (coded as striped or unstriped), we segmented each individual salamander from the batch photographs. This was done via a script that detected color and edge, then set a convex hull around each salamander which was then used to cut individual specimens from the original image (Fig. 1). Using hulls before cutting provided a means of spot checking the success of image segmentation approaches.

After segmentation, individual images were uploaded to the web-based community science platform, Notes from Nature (NFN^52^). Volunteers were asked to record the collection identification number (i.e. tag number) and indicate the color morph (striped or unstriped) of each individual salamander. Scoring was done in triplicate to account for human identification errors. Classification of salamander color morphs by community scientists on NFN took approximately five weeks. With reconciled data from the NFN project, we identified the majority-rule (2/3 scored the same) morph type, after checking a subset of scores to assure that results were generally accurate. The recorded tag numbers from the NFN project were cross-referenced with VertNet (vertnet.org^53–55^) to obtain full specimen records (e.g. date, latitude, and longitude). Due to duplicated images and a few mismatched transcriptions of specimen tag numbers, the resulting dataset included 2,862 records (96% of the imaged specimens) of *P. cinereus*.

### Climate and elevation data

We obtained historical climate data for georeferenced localities using ClimateNA at 4 km resolution^56^. Using default settings, we extracted mean annual temperature (MAT) and mean annual precipitation (MAP) based on the latitude, longitude, and year of collection for each salamander. We used the ‘get_elev_point’ function in the R package *elevatr*^57^ to obtain elevation data based on specimen latitude and longitude.

### Statistical analyses

#### Morph frequency

Our first set of models tested whether the ratio of the two color morphs varies with climatic variables and if morph frequency changed over time. To examine these relationships, we ran a logistic regression using R base glm() function^58^ with a binomial family and logit link function. The response variable was coded 1 for striped and 0 for unstriped morphs and we used six fixed predictors: year of collection, MAT, MAP, season of collection (spring, summer, fall, winter), elevation, and mitochondrial clade. Radomski et al.^50^ found strong support for high levels of genetic structure among these groups (clade names correspond with Radomski et al.^50^; Northern, Virginia, Southern; Fig. 2). Given limited random-effect levels, we did not attempt to nest site-level effects within clade.

#### Body size

We ran linear models (LM) via R’s base lm() function^58^ to examine body size trends in *P. cinereus*. First, we removed juveniles from this dataset to reduce bias. Our size cut-off for adults was a SVL of 34 mm^24^, resulting in a dataset of 2131 records. Our global model included adult body size (SVL) as the response variable and the predictors were: color morph, year, MAT, MAP, season of collection (spring, summer, fall, winter), elevation, and mitochondrial clade. To account for divergent changes in SVL by color morph, we modeled morph interactions with each predictor: morph x year, morph x MAT, morph x MAP, morph x season, morph x elevation, and morph x clade.

For both morph frequency and body size models, all continuous predictors were mean-centered and scaled, and categorical predictors were transformed to factors. All analyses were conducted in R 3.6.1^58^. We used the ‘dredge’ function in the R package *MuMIn*^59^ to rank and assess the best fit morph frequency and body size models with AICc. We ran a logistic regression and linear model on the best fit models of color morph frequency and body size, respectively, to examine significant fixed effects.

### Ethical approval

All salamander specimens were preserved and were accessed in person with permission from staff at the NMNH.

## Results

### Morph frequency models

**Figure 3:**
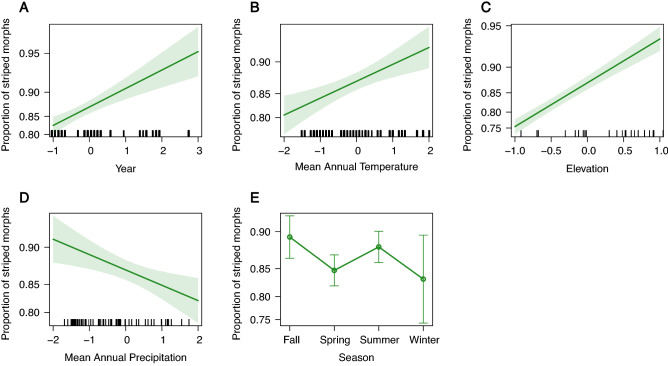
Top model effect plots of significant predictors of color morph frequency in *Plethodon cinereus*. The proportion of color morphs is influenced by (**A**) year, (**B**) mean annual temperature, (**C**) elevation, (**D**) mean annual precipitation, and (**E**) season. Error bars in each plot represent 95% confidence intervals.

The top model of morph frequency variation included MAT, MAP, year, elevation, and season (Table 1, S1). The proportion of striped morphs has increased over time (*β* = 0.35, *SE* = 0.07, *p* < 0.001; Fig. 3A) and was positively correlated with both MAT (*β* = 0.25, *SE* = 0.07, *p* < 0.001; Fig. 3B) and elevation (*β* = 0.79, *SE* = 0.07, *p* < 0.001; Fig. 3C). Striped morph frequency was negatively correlated with increased MAP (*β* =  − 0.19, *SE* = 0.07, *p* = 0.004; Fig. 3D). There is a decreased proportion of striped morphs in the spring compared to the fall, but there is no difference for any other season (spring-fall *β* =  − 0.42, *SE* = 0.16, *p* = 0.009; summer-fall *β* =  − 0.12, *SE* = 0.16, *p* = 0.452; winter-fall *β* =  − 0.53, *SE* = 0.31, *p* = 0.092; Fig. 3E).

**Table 1:** Top (A) color morph frequency and (B) body size (SVL) model results.

Term	Estimate	Std. Error	*p* value
(A) *Color morph*			
**Intercept**	**2.129**	**0.135**	**< 0.001**
**Year**	**0.354**	**0.074**	**< 0.001**
**MAT**	**0.246**	**0.067**	**< 0.001**
**MAP**	− **0.191**	**0.066**	**0.004**
**Elevation**	**0.791**	**0.069**	**< 0.001**
**Season:spring**	− **0.416**	**0.158**	**0.009**
Season:summer	− 0.123	0.164	0.452
Season:winter	− 0.525	0.312	0.092
(B) *Body size*			
**Intercept**	**41.525**	**0.318**	**< 0.001**
**Morph:unstriped**	**1.462**	**0.510**	**0.004**
**Year**	− **0.337**	**0.089**	**< 0.001**
**MAT**	− **0.875**	**0.103**	**< 0.001**
Elevation	0.284	0.232	0.222
**Season:spring**	− **0.737**	**0.254**	**0.004**
**Season:summer**	− **1.823**	**0.260**	**< 0.001**
Season:winter	0.777	0.426	0.068
Clade:southern	0.528	0.480	0.272
**Clade:virginia**	**1.624**	**0.441**	**< 0.001**
**MAT × morph:unstriped**	**0.686**	**0.247**	**0.005**
**Year × morph:unstriped**	**1.319**	**0.305**	**< 0.001**
**Elevation × morph:unstriped**	**1.097**	**0.281**	**< 0.001**
**Season:spring × morph:unstriped**	− **1.471**	**0.608**	**0.016**
Season:summer × morph:unstriped	− 0.598	0.619	0.335
Season:winter × morph:unstriped	− 0.490	1.009	0.627

Bold effects are significant.

### Body size models

**Figure 4:**
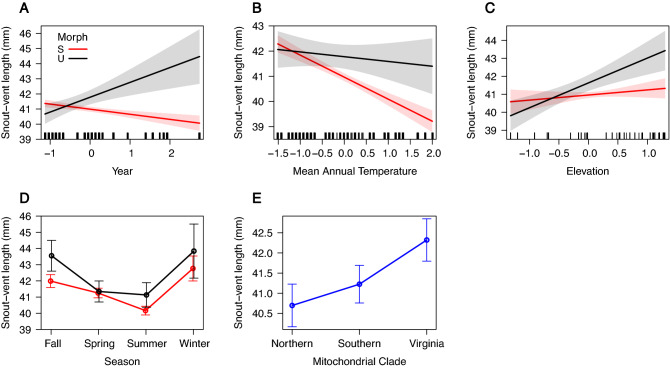
Top model effect plots of significant predictors of body size variation in *P. cinereus*. Body size (snout–vent length) is influenced by (**A**) the interaction between color morph and year, (**B**) the interaction between color morph and MAT, (**C**) the interaction between color morph and elevation, (**D**) the interaction between color morph and season, and (**E**) mitochondrial clade. Error bars in each plot represent 95% confidence intervals.

The best fit model of variation in SVL included the covariates morph, MAT, season, year, elevation, clade, and the interactions of morph x year, morph x MAT, morph x season, morph x elevation (R^2^ = 0.121, Table 1, S1). Striped morphs decrease in SVL over time, whereas unstriped morphs increase in size (*β* = 1.32, *SE* = 0.31, *p* < 0.001; Fig. 4A). Striped and unstriped morphs decreased in SVL with increasing MAT, but the strength of the decrease was stronger for striped morphs (*β* = 0.67, *SE* = 0.25, *p* = 0.005; Fig. 4B). Both striped and unstriped morph body size increased with increasing elevation, but the strength of the increase was stronger for unstriped morphs (*β* = 1.10, *SE* = 0.28, *p* < 0.001; Fig. 4C). Body size of surface-active striped *P. cinereus* morphs are smaller in the spring, compared to unstriped morphs in the fall, with no other morph-related differences among seasons (spring-fall *β* =  − 1.47, *SE* = 0.61, *p* = 0.016; summer-fall *β* =  − 0.60, *SE* = 0.62, *p* = 0.335; winter-fall *β* = 0.49, *SE* = 1.01, *p* = 0.627; Fig. 4D; Table 1). Regardless of color morph, salamanders do not differ in size between the Southern and Northern clade, but are larger in the Virginia clade compared to the Northern clade (Southern-Northern, *β* = 0.53, *SE* = 0.48, *p* = 0.272; Virginia-Northern, *β* = 1.62, *SE* = 0.44, *p* < 0.001; Fig. 4E).

## Discussion

Species, especially those with narrow niche requirements, are expected to respond to climate change either by shifting phenology, range distributions, or by plastic or adaptive phenotypic responses^2,60–63^. We expected changing climate to promote evolutionary morphological change in the polymorphic salamander, *P. cinereus*, especially given its low vagility. We also expected these changes to be conditioned by color morph, based on evidence of niche separation between the morphs^24^. Our results from a set of time series data, covering over four decades of sampling and encompassing multiple known geographically structured populations, confirms a phenotypic response—albeit, not in the predicted direction—with striped morph frequency increasing in relation to climate warming and through time. Further, we found morph-specific body size responses to climate whereby the temporal effect is decreased size of the striped morph and increased size of the unstriped morph.

Our results show that temperature, precipitation, and elevation influence the proportion of morphs found above-ground over time, in contrast to previous studies that found no correlation between color morph frequency and these variables in *P. cinereus*^25,41^. While color morph frequency follows a predicted response to elevation (i.e. increasing striped morph frequency with increasing elevation), our climate-related findings do not match initial predictions on the direction of these relationships. Instead, we found the proportion of striped morphs increased with temperature and decreased with precipitation. Past ecological studies suggested that the striped morph is more common in cooler and wetter habitats than the unstriped morph^34,38,39^. The unstriped morph has a lower metabolic rate, facilitating increased activity in warmer, drier conditions^33,36,64^. These previous studies are largely restricted to single populations at single points in time and do not account for selection pressures that may differ across space and time^65–68^ in wide-ranging species like *P. cinereus*. It is probable that morph frequency is a key element of a complex spatial mosaic of coevolutionary processes^69,70^. Further discrepancies may relate to our use of a hierarchical modeling framework, which, in addition to climate, also accounts for phylogenetic structure and elevation. Previous studies documented a weak, but positive association between elevation and frequency of the striped morph^25,39^. Here, we find a stronger positive relationship between the proportion of striped morphs and elevation, suggesting that the effects of temperature and elevation are decoupled in relation to color morph frequency in this species.

One possible explanation for our unexpected results for climate-related morph frequency is that warming per se is not a direct effect. Instead, warming and precipitation effects indirectly mediate body size. Red-backed Salamanders are nocturnal thermoconformers and thus do not experience direct UV-radiation that may influence whether more melanistic forms (i.e. unstriped morph) experience overheating more often than paler forms as in other ectotherms^71^. The decrease in the proportion of striped morphs with increased precipitation may suggest instead that these salamanders follow Gloger’s Rule (i.e. animal coloration is darker in more humid areas and paler in drier environments^72^). Although the mechanistic basis of this ecogeographic rule remains unclear, melanistic animals may be more camouflaged in humid environments where dense vegetative cover and rich organic soils offer low light conditions and darker background colors, reducing predator detection^73,74^. Hantak and Kuchta^75^ investigated morph camouflage in *P. cinereus* and found striped morphs were better camouflaged than unstriped morphs against most background types; however, the degree of camouflage was dependent on locality, season, and predator type. It may be that simplistic, direct links between climate and morph do not explain why we see an increase in the proportion of striped morphs over time. Instead, morphs may be responding to changes in climate through co-adapted changes in morphology.

Shifts in body size is a key component mediating the effects of climate change^62^*.* Based on previous ecological studies, we predicted a decrease in size for striped morphs but no change for unstriped morphs in response to climate. Our results show instead that both striped and unstriped morphs respond to warming temperatures with decreases in body size, but the striped morph is more sensitive to increased temperatures. The temperature-size rule states that warmer temperatures should increase developmental rate in ectotherms, leading to smaller body size^76^. However, this does not account for increased sensitivity of striped morphs to warmer temperatures. With a higher proportion of striped morphs on the surface in warmer temperatures, selection on body size may be stronger for this morph. Increased exposure of the striped morph to warmer temperatures could result in smaller size, as a selective response, compared to the unstriped morph^77^. Because morphs are composed of co-adapted trait complexes, it is probable that correlational selection has acted on a suite of traits (e.g. body size and color pattern) to produce differentially adapted character sets^14^, ultimately allowing striped morphs to be more active on the surface in warmer conditions.

In contrast to the temperature-related findings, both color morphs increased in body size with increasing elevation, but unstriped morphs increased more than striped morphs. Previous studies have generally documented intraspecific increases in size for plethodontid salamanders at higher elevations^78–80^. Why the unstriped morph exhibits a stronger positive relationship between size and elevation is unknown. It is possible that physiological differences between the morphs play a role in the association between elevation and body size. For instance, variation in evaporative water loss and metabolic rate across elevational gradients may result in body size differences between morphs^80^. Metabolic rate appears to be co-adapted with color in *P. cinereus*, with the unstriped morph generally exhibiting a lower metabolic rate; however, this trend is not consistent across seasons and localities^33,36^. No studies have examined physiological differences between morphs along elevational gradients, but this work would provide more information on the relationship between color morph and body size in this species.

Finally, our results show a pattern of striped morphs becoming shorter over time, while unstriped morphs are getting longer. Reductions in body size of the striped morph over time aligns with our findings of temperature-size interactions, but the increased size of the unstriped morph was unexpected. Previous studies have suggested that the unstriped morph contains more trunk vertebrae than the striped morph, likely related to increased fossoriality^64,81,82^. We predicted unstriped morphs would show no overall change in body size because increased time underground would allow this morph to avoid unfavorable climatic conditions. Why is the unstriped morph increasing in size over time, especially given the decreasing size of this morph in warmer temperatures? It is possible that warmer temperatures lead to increased fossoriality of the unstriped morph^64^, an interpretation consistent with decreased frequency of surface-active unstriped morphs in warmer temperatures and over time. However, the environmental conditions that lure the larger unstriped morphs to the surface remain unclear. Further work examining body size variation between color morphs would benefit from evaluation of surface area to volume ratios of morphs^83^, in addition to trunk vertebrae counts, which is the known mechanism of elongation in *Plethodon*^84^. We collected digital x-rays for a small subset of salamanders included in our study, and these data indicate that counting vertebrae is a feasible next step for further exploring the divergence in body size between the morphs.

Examining morph-specific behavioral responses to climate warming may provide information on the degree of niche separation between the morphs. Thermal buffering appears to be a key life history trait mediating the effects of climate in the unstriped morph, given its increased fossoriality. Temperature may act as a stronger pressure on body size for the striped morph if its thermal buffering ability is more limited^77^. Future studies testing behavioral variation in the time spent above or below ground would provide information on whether the unstriped morph is more fossorial. In addition, the two morphs of *P. cinereus* are likely utilizing different microclimates (e.g. soil temperature and moisture) to avoid direct effects of ambient air temperature^85^. Our study was conducted at a relatively course-scale of climate resolution (4 km) for plethodontid salamanders; thus, finer-scale studies of niche separation between the morphs will help further elucidate how the morphs cope with climate change. Finally, although our dataset is limited by past collection efforts, there is no indication of morphological bias in the collected salamanders. In addition, our modeling approach accounts for seasonal variation in salamander detections, and while sample sizes across collection dates and years vary, we see no systematic biases over season or year^86^.

Our work complements and extends previous efforts in other systems showing that changing climatic conditions alters color morph frequencies (e.g., birds^71,87^; reptiles^88^; fish^89^; insects^90^; and plants^91^). Declining frequency or complete loss of a color morph can change the local selective environment^15^. For instance, loss of a Side-blotched Lizard (*Uta stansburiana*) color morph resulted in rapid phenotypic change in body size of the remaining morphs, likely as a result of alterations in the local competitive environment^70^. Apart from the current study, climate-related changes in co-adapted morphological traits have been underexplored despite this variation likely playing a role in species divergence^15,92^. Further work investigating the impacts of climate on co-adapted traits in polymorphic species is still needed to better elucidate relationships between color polymorphism and diversity.

## Conclusions

Determining phenotypic responses of species to climate change is constrained by acquiring data over longer timescales. Using a historical dataset, we examined the response of two measures of phenotypic change in relation to climate in the most abundant terrestrial vertebrate in the northeastern United States, the color polymorphic salamander, *P. cinereus*^22^. Color polymorphic species serve as useful models for response to warming because these species may be less vulnerable to environmental change as co-adapted trait combinations allow morphs to cope with rapid changes in climate^16^. As long as gene flow persists between morphs, color polymorphisms may be maintained by divergent selection stemming from niche partitioning, which allows morphs to occupy novel environments^93,94^. Hantak et al.^95^ found low to moderate levels of gene flow across sites that varied in color morph frequency of *P. cinereus*. Our current study demonstrates that color morphs of *P. cinereus* differentially respond to climate through changes in the distribution or surface activity of the morphs and body size. The proportion of striped morphs increases with warmer temperatures, elevation, and through time but decreases with increasing precipitation. Both morphs demonstrate body size decreases in warmer temperatures, but the striped morph is more sensitive to changes in temperature. Conversely, both morphs show body size increases at higher elevations, but the strength of the increase is stronger for unstriped morphs. Finally, we found reductions in striped morph size over time but increased size for the unstriped morph. In addition to *P. cinereus* there are nine other species within the genus *Plethodon* that display the striped/unstriped color polymorphism, and closely related species are typically fixed for either phenotype. We know little about these other polymorphic species of *Plethodon* and whether they too show similar co-adapted trait complexes (but see^44^). Future work on this color polymorphism in *Plethodon* will benefit from comparative studies across the genus.

## Supplementary Information

Supplementary Table S1.

## Data Availability

Data and code for this study are available at https://github.com/mhantak/Plethodon_cinereus_temporal.
