# Supplementary material for: Rapid phenotypic change in a polymorphic salamander over 43 years
Source: Sci Rep. 2021 Nov 22;11:22681. doi: 10.1038/s41598-021-02124-2 (PMC8609024; doi:10.1038/s41598-021-02124-2)
Supplement: Supplementary file 1 — Supplementary Table S1. [file 41598_2021_2124_MOESM1_ESM.docx]

**Rapid phenotypic change in a polymorphic salamander over 43 years**

Maggie M. Hantak^1*^, Nicholas A. Federico^1^, David C. Blackburn^1^, Robert P. Guralnick^1^

^1^Florida Museum of Natural History, University of Florida, Gainesville, FL, USA

*Correspondence: Maggie Hantak, Email: maggiehantak@gmail.com

**SUPPLEMENTAL TABLE**

Table S1. Top five models of (A) color morph frequency and (B) body size for *Plethodon cinereus*. Direction of continuous predictor estimates are provided (+ = positive; - = negative).

| **Model** | **df** | **AICc** | **ΔAICc** | **AICcWt** |
| --- | --- | --- | --- | --- |
| **(A) Color morph frequency** |  |  |  |  |
| MAT (+), MAP (-), Year (+), Elevation (+), Season | 8 | 2267.1 | 0.00 | 0.588 |
| MAT (+), MAP (-), Year (+), Elevation (+), Clade, Season | 10 | 2269.7 | 2.61 | 0.160 |
| MAT (+), MAP (-), Year (+), Elevation (+) | 5 | 2269.8 | 2.76 | 0.148 |
| MAT (+), MAP (-), Year (+), Elevation (+), Clade | 7 | 2272.8 | 5.73 | 0.034 |
| MAT (+), Elevation (+), Year (+) | 4 | 2273.5 | 6.44 | 0.023 |
| **(B) Body size** |  |  |  |  |
| MAT (-), Morph, Year (-), Elevation (+), Clade, Season, Morph:MAT, Morph:Year, Morph:Elevation, Morph:Season | 17 | 11238.0 | 0.00 | 0.174 |
| MAT (-), Morph, Year (-), Elevation (+), Clade, Season, Morph:MAT, Morph:Year, Morph:Elevation, | 14 | 11238.2 | 0.25 | 0.154 |
| MAT (-), MAP (+), Morph, Year (-), Elevation (+), Clade, Season, Morph:MAT, Morph:Year, Morph:Clade | 15 | 11239.6 | 1.59 | 0.079 |
| MAT (-), MAP (+), Morph, Year (-), Elevation (+), Clade, Season, Morph:MAT, Morph:Year, Morph:Elevation, Morph:Season | 18 | 11239.8 | 1.83 | 0.070 |
| MAT (-), Morph, Year (-), Clade, Season, Morph:MAT, Morph:Year, Morph:Clade | 14 | 11240.0 | 2.00 | 0.064 |
